# Proteomics tools reveal startlingly high amounts of oxytocin in plasma and serum

**DOI:** 10.1038/srep31693

**Published:** 2016-08-16

**Authors:** Ole Kristian Brandtzaeg, Elin Johnsen, Hanne Roberg-Larsen, Knut Fredrik Seip, Evan L. MacLean, Laurence R. Gesquiere, Siri Leknes, Elsa Lundanes, Steven Ray Wilson

**Affiliations:** 1 Department of Chemistry, University of Oslo, Post Box 1033, Blindern, NO-0315 Oslo, Norway; 2 School of Pharmacy, University of Oslo, PO Box 1068, Blindern, NO-0316, Oslo, Norway; 3 Department of Evolutionary Anthropology, Duke University, Durham, NC, 27708, USA; 4 School of Anthropology, University of Arizona, Tucson, AZ, 85721, USA; 5 Department of Biology, Duke University, Durham, NC, 27708, USA; 6 Department of Psychology, University of Oslo, PO Box 1094, Blindern, NO-0317, Oslo Norway; 7 The Intervention Centre, Oslo University Hospital, PO Box 4950, Nydalen, NO-0424 Oslo, Norway

## Abstract

The neuropeptide oxytocin (OT) is associated with a plethora of social behaviors, and is a key topic at the intersection of psychology and biology. However, tools for measuring OT are still not fully developed. We describe a robust nano liquid chromatography-mass spectrometry (nanoLC-MS) platform for measuring the total amount of OT in human plasma/serum. OT binds strongly to plasma proteins, but a reduction/alkylation (R/A) procedure breaks this bond, enabling ample detection of total OT. The method (R/A + robust nanoLC-MS) was used to determine total OT plasma/serum levels to startlingly high concentrations (high pg/mL-ng/mL). Similar results were obtained when combining R/A and ELISA. Compared to measuring free OT, measuring total OT can have advantages in e.g. biomarker studies.

The neuropeptide oxytocin (OT) is a facilitator of childbirth and breastfeeding, and can activate maternal behavior^1^ and partner preference^2^ in animal models. In humans, OT levels have been related to e.g. autism^3^, and schizophrenia^4^. Several studies have reported a coordinated release of central and peripheral OT^5^,^6^ and that peripheral levels can provide a minimally-invasive indicator of central state^7^. However, OT measurements (and interpretation of these) are often met with skepticism. Nearly without exception, enzyme-linked immunosorbent assays (ELISA) and radioimmunoassays (RIA) are used to monitor OT in blood and other biofluids; the selectivity of these methods is criticized^8^,^9^. An alternative to ELISA/RIA is mass spectrometry (MS)^10^,^11^,^12^. The MS instrument allows unambiguous identification/quantification of e.g. peptides, by first recording the molecular mass of a compound (single MS), and then creating a “molecular fingerprint” by fragmenting the compound to smaller parts (MS/MS). Separating compounds in a mixture (e.g. plasma) prior to MS detection further strengthens identification and sensitivity. Peptides are typically separated using liquid chromatography (LC). LC-MS is an invaluable tool in virtually all areas of biomedical analysis. A notable exception however is OT measurement in blood; the few published methods for LC-MS measurements of plasma OT^11^,^12^ provide unsatisfactory sensitivity and varying results, and are therefore difficult to put to practical use. We here set out to develop a robust and sensitive method for quantification of OT in blood. We here “borrow” tools from mass spectrometry based proteomics, namely nanoLC-MS (a particularly sensitive variant of LC-MS^13^) featuring on-line sample extraction^14^, and a reduction/alkylation step^15^ that allows also the protein-bound fraction to be measured. We also apply a lipidomics nanoLC plumbing scheme^16^,^17^ to enable very robust targeted peptidomics.

## Results

### Enabling nanoLC-MS for robust and simple plasma analysis

**Figure 1:**
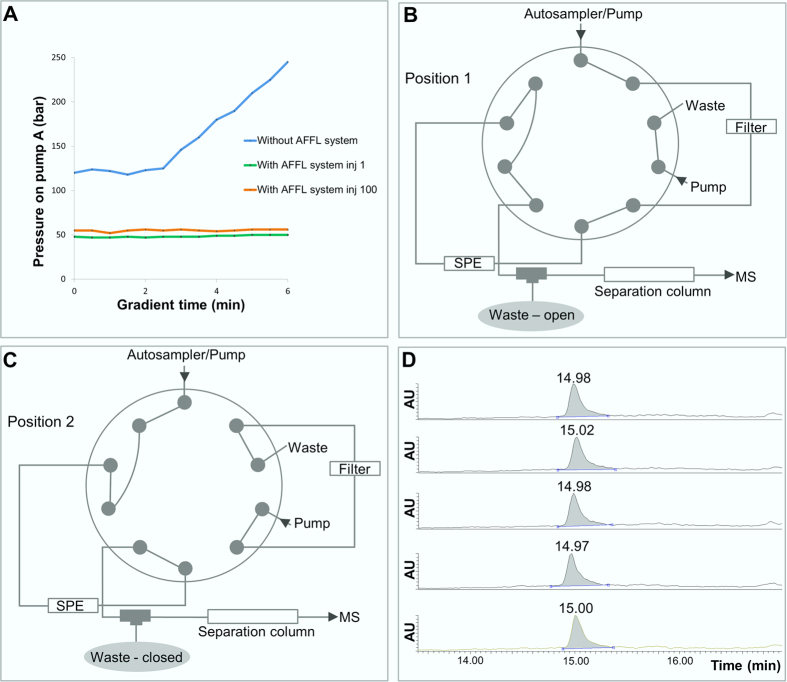
AFFL-SPE-nanoLC-MS for plasma analysis. (**A)** Pressure profiles on pump A of the Easy nLC pump, during a 6 minutes gradient (0–90% B) at a flow rate 800 nL/min, when injecting a plasma sample without the AFFL system (blue line). The green line illustrates the pressure profile during the gradient for the first plasma sample injected when the AFFL system was incorporated, while the orange line illustrates the pressure profile during the gradient for the hundredth plasma injection onto the AFFL system. (**B)** Position 1 of the external 10-port valve. In this position the particles are retained on the filter, while hydrophobic compounds (including OT) is retained on the SPE and salts and hydrophilic compounds are eluted to waste. (**C)** Position 2 of the external 10-port valve. The filter is being back-flushed, and hydrophobic compounds are eluted off the SPE and separated on the separation column before detection by MS. (**D)** Five injections of plasma sample spiked with OT to a final concentration of 500 pg/mL.

In preliminary experiments with a standard nanoLC-MS set-up (i.e. trap column for extraction + separation column), injecting protein precipitated pooled human plasma clogged the column(s) (see Fig. 1A). After just one plasma injection, it was not possible to reuse the columns, even after extensive washing attempts. Therefore, we incorporated an AFFL system^16^,^17^ upstream to the nanoLC-MS platform (see Fig. 1B,C). AFFL allows samples to pass through a stainless steel filter that captures particulate matter; this matter is flushed backwards off the filter after each injection, allowing filter intactness (and hence system robustness) for very large numbers of injections. To illustrate, only a minimal increase in back pressure between the first to the hundredth plasma injection was observed (Fig. 1A). This result mirrored a similar experiment, performed with cell lysate samples^17^. OT spiked to plasma could be chromatographed with excellent retention time repeatability (0.1% RSD; see Fig. 1D). During this study, over 300 samples were injected without need for part/column replacement. Silica monolith nano LC columns^18^ provided stable and efficient resolution. Taken together, AFFL-SPE-nanoLC-MS is a highly suited platform for blood peptidomics, e.g. targeted determination of oxytocin.

### Sensitive and stable detection of plasma OT following a reduction/alkylation step

We find that OT strongly binds to plasma proteins. Performing a reduction and alkylation step liberates OT from plasma proteins, allowing ample sensitivity and precise quantification of endogenous (total) OT. Details are described below.

Initially, samples contained 50 mM ZnCl_2_ (10 mM aspartate buffer, pH 4.5) to stabilize OT via chelation^19^ prior to subsequent sample preparation (e.g. removing proteins via protein precipitation (PPT). However, adding ZnCl_2_ to plasma samples resulted in noisy signals and pressure build-up, likely due to on-column precipitation of salts and/or proteins. Acetonitrile based PPT (without the presence of chelating agents) was associated with an unassuring recovery profile (OT recovery dropped and leveled off after 40 minutes (Figure SM1)). OT was stable in the solvents used during and after PPT (Figure SM2), and did not stick to tubes and vials. It was considered unlikely that the main metabolizing enzyme for OT in plasma, cystinyl aminopeptidase/oxytocinase^20^ was degrading OT in these conditions, as this enzyme is rather large (subject to PPT), and blood from non-pregnant individuals was used. Therefore, we speculated that the recovery profile depicted a slow binding to protein remains. To further assess the issue of OT protein binding, pooled human plasma was spiked with oxytocin, and was stored on the laboratory bench up to 8 h before PPT; recovery of the spiked OT linearly deteriorated as function of time before the PPT step (Figure SM3), once again suggesting a slow and strong protein binding after spiking. Furthermore, OT spiked to plasma had very poor filtrate recovery using size separation with centrifugal filters, again implying strong protein binding.

**Figure 2:**
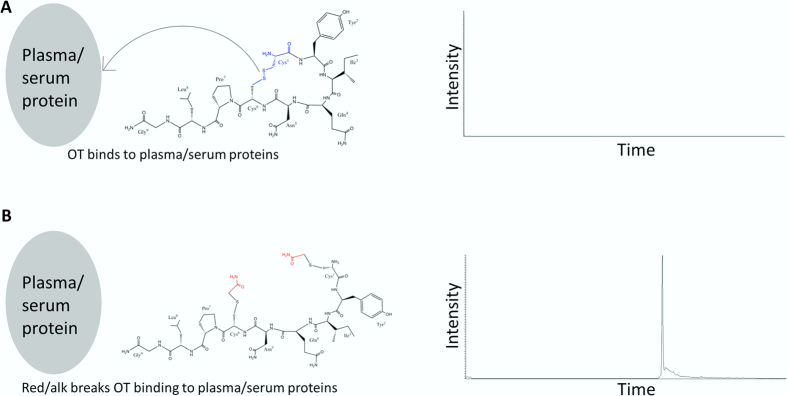
Effect of reducing and alkylation on oxytocin measurement in plasma. (**A)** OT binds to plasma/serum proteins, and co-precipitate during sample preparation, resulting in poor detection. (**B)** Reduction and alkylation breaks OT binding to plasma/serum proteins, preventing loss of oxytocin during sample preparation, resulting in ample detection.

We hypothesized that strong protein binding was preventing detection of endogenous OT (Figure SM4) due to co-precipitation during PPT. The disulfide bridge (DSB) of OT (Fig. 2A) can engage in complexes^19^, and likely with serum albumin, which contains multiple DSBs. To obstruct plasma protein binding, a reduction/alkylation (R/A)^15^ step was performed which irreversibly breaks DSBs (Fig. 2A). Advantages of performing R/A of oxytocin to obtain stable species have been reported/implied^21^. Complete and stable derivatization was achieved; no native spiked OT was detected after R/A treatment (results not shown). When analyzing unspiked R/A treated plasma samples, total endogenous OT was found to be present at strikingly high levels (see LC-MS chromatogram, Fig. 2B and Figure SM5).

**Figure 3:**
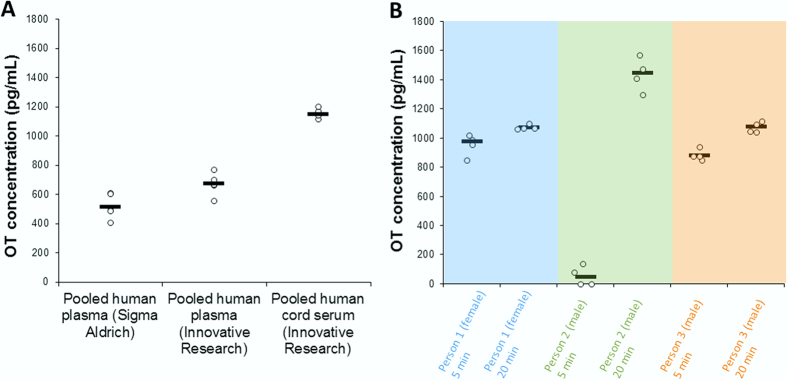
Oxytocin levels in human plasma/serum. (**A)** OT basal levels in pooled human plasma from two vendors (Sigma Aldrich and Innovative Research), and OT basal levels in pooled human cord serum from Innovative Research. (**B)** OT plasma concentration from three subjects after 5 and 20 minutes after applying two puffs of OT nasal spray in each nostril.

Total OT was determined in pooled plasma and human cord serum (from approximately 20 persons), obtained from commercial sources: The concentration of oxytocin in pooled human plasma from Sigma Aldrich and Innovative Research was 0.5 ng/mL and 0.7 ng/mL, respectively. For pooled human cord serum (Innovative Research) the OT concentration was expectedly higher^22^, 1.2 ng/mL (Fig. 3A). Oxytocin plasma levels were, as expected, higher after nasal intake of OT (Fig. 3B). However, the fold-change was very dependent on the individual. For instance, person 2 (who self-reported high levels of anxiety prior to sample collection) had a markedly different OT plasma profile. Our results confirm the common assumption that OT levels can significantly vary between individuals^23^, and we expect that the OT levels in the samples analyzed in this initial study will not be representative of all individuals, conditions or sample handling procedures. Interference-free identification/quantification of OT was based on using external standards, a deuterated internal standard, and characteristic MS/MS transitions for quantification/qualification. The quantitative traits of the assay included excellent linearity (5–2000 pg/mL spiked to same-batch plasma, r^2^ = 0.999), high recovery (90%) and good precision/reliability (RSD: 0.4–4.3%, depending on concentration); see Figure SM6. Within-sample variations can occur (Fig. 3) if samples are somewhat inhomogeneous (due to e.g. variance in the efficiency of red blood cell removal for obtaining plasma), calling for performing several replicates per sample (if possible).

## Discussion

A reduction and alkylation step was key in stably “liberating” oxytocin from plasma proteins, allowing ample detection of endogenous high pg-ng/mL amounts in human plasma. Tight plasma binding is not uncommon with biomarkers^24^. The OT levels observed in this study are several orders higher compared to that obtained with an off-line extraction step + ELISA/RIA^25^, but more in agreement with an approach involving an isolation of redox sequestered fractions in plasma^26^. With extraction only, the vast majority of OT is discarded with plasma proteins, leaving only a minute free amount of OT left to be measured (previously believed to represent the majority of OT). Measuring only the free fraction, as currently recommended^8^ can in many cases be a confounding factor, since the free OT concentration can be drastically changed by factors such as age, morbidity, or by compounds/drugs that displace OT from proteins^27^. This is especially the case if the marker is heavily bound^27^, as we find with OT. Indeed, large variations are observed when measuring the free fraction of OT; a third of the human samples analyzed by Zhang *et al*. (MS approach) did not contain detectable levels of OT^11^. We have also registered such inconsistencies with our own “neurotransmitter-omics” MS platform^12^. In addition, free OT levels varied 6-fold within a homogenous group of rats^11^. As shown in Fig. 3, when all circulating OT is measured the differences between individuals are already pronounced (but not unusually large compared to much of the metabolome). Such individual differences are thought to be highly informative^23^,^28^; additional confounding factors will however make correlations less clear. Based on this reasoning, total OT may in many cases be a better suited as a biomarker than the free fraction of OT. However, the biological activity of the bound fraction is less clear, and subsequent studies should be performed to investigate this.

**Figure 4:**
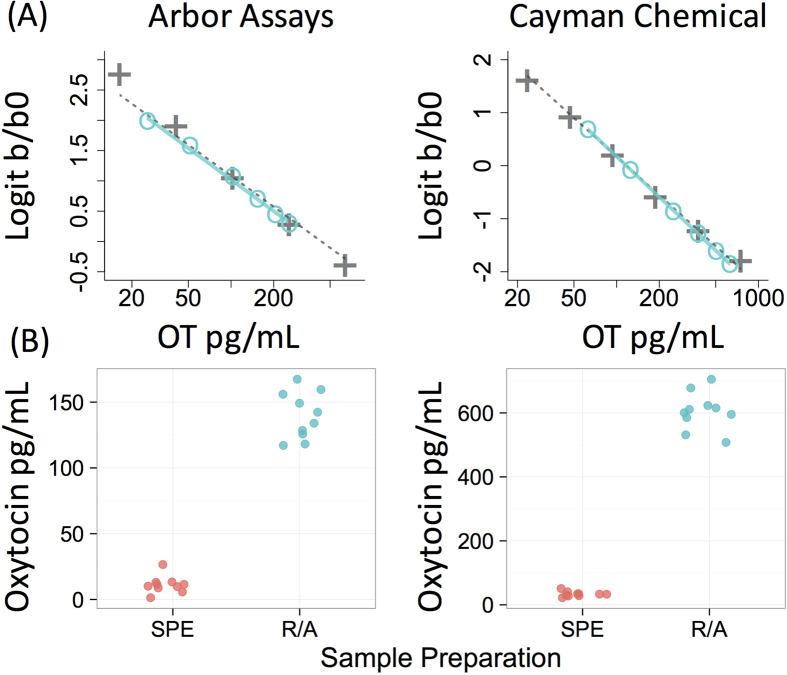
Parallelism for ELISA using reduction/alkylation treated plasma and comparison to solid phase extraction. (**A)** Kit standards (gray + ’s) and a series of plasma dilutions (turquoise circles) processed using reduction/alkylation (R/A) and protein precipitation (PPT). Parallelism and regression fits predicting observed from expected values were excellent for both kits (Arbor Assays: β = 0.98, R^2^ = 1.0; Cayman Chemical: β = 1.0, R^2^ = 0.99). (**B)** Oxytocin concentrations in 10 plasma samples measured following solid phase extraction (SPE) or R/A and PPT. For both kits R/A led to a dramatic increase in detectable oxytocin.

The R/A approach applies to other detection techniques/samples; two commercially available ELISA kits revealed large increases in detectable OT of dog plasma using this approach (Fig. 4). In contrast to neat plasma, R/A treated plasma also yielded excellent linearity and parallelism with both kits (Fig. 4). However, the concentrations of OT detected using ELISA varied between kit manufacturer, implying differences in selectivity, and therefore it will be important for future studies to benchmark the battery of ELISA assays available against mass spectrometry. Also related to selectivity, it is unlikely that the reduced form of OT, oxytoceine, is being measured simultaneously as the reduced/alkylated form, as oxytoceine is not stable in aqueous, neutral solutions (e.g. blood)^29^.

LC-MS can be considered a natural choice for OT measurements due to its excellent selectivity. The robust and highly automated AFFL-nanoLC-MS, i.e. on-line sample filtration, enrichment and separation (R/A and PPT steps performed off-line), approach has attractive quantification traits, and can be simply implemented in e.g. proteomics core facilities (common in e.g. many larger universities/hospitals). Only 100 μL plasma is needed per measurement, leaving sufficient amounts of a 5–10 mL blood sample available for other analyses. Other LC-MS systems can be employed, e.g. UPLC-MS systems used for drug measurements or metabolomics, but these may require off-line filtration/extraction steps.

## Methods

### Chemicals and reagents

Oxytocin (OT) acetate salt hydrate (≥97%), oxytocin-d_5_ (98%, internal standard (IS)), dithiothreitol (DTT), iodoacetamide (IAM), acetonitrile (LC-MS grade), formic acid (FA, LC-MS grade) and pooled human plasma with 4% trisodium citrate as anticoagulant (P9523-5 mL, lot#: SLBK0464V) were purchased from Sigma Aldrich (St. Louis, MO, USA). Pooled human plasma with EDTA as anticoagulant (lot#: 17964) and pooled human cord serum (lot#: 18241) were obtained from Innovated Research (Huntsville, AL, USA). 1 M Tris-HCl pH 8.0 was made by Oslo University Hospital (Oslo, Norway). LC-MS grade water was bought from Fischer Scientific (Hampton, NH, USA), while type 1 water was acquired from a Milli-Q Integral 5 water purification system (Merck Millipore, Billerica, MA, USA).

### Storage of stock solutions, plasma and serum

Stock solutions of OT (5 μg/mL) and IS (10 μg/mL) dissolved in LC-MS grade water, pooled human plasma and pooled human cord serum were stored in freezer at −20 °C.

### Statement

All experiments and methods were performed in accordance with relevant guidelines and regulations. All experimental protocols were approved by a named institutional/licencing committee. Specifically, human blood collection and nasal spray experiments (and relevant protocols) were approved by the Regional Ethics Committee (REC) (2011/1337/REK S-OE D) (Oslo, Norway). Informed consent was obtained from all subjects, and all methods were carried out in accordance with the relevant guidelines and regulations of REC. Dog blood collection was approved by the Institutional Animal Care and Use Committee (IACUC) at Duke University, and experiments (and relevant protocols) were performed/used in accordance with the relevant guidelines and regulations of IACUC Duke University, Protocol # A112-14-10.

### Preparation of calibration standards and samples

For all standard solutions and plasma/serum samples, 10 μL of a 10 ng/mL working solution of IS were added so that the concentration in the final reconstitution volume (100 μL) was 1 ng/mL IS. All solutions were made in 1.5 mL Eppendorf LoBind tubes (Hamburg, Germany). Standard solutions used for establishing the calibration curve were made by appropriate diluting a working solution of 10 ng/mL OT in 0.1% FA with 0.1% FA to a final concentration in the reconstituted solutions of 5, 500, 1000 and 2000 pg/mL. Dilution of the plasma/serum samples and standard solutions was performed by pipetting (with newly calibrated pipettes) 100 μL of plasma/serum samples and standard solutions into 200 μL 50 mM tris-HCl (pH 8.0). For reduction of disulfide bonds, 5 μL of 0.5 M DTT were added to all solutions followed by whirl mixing for 30 sec, incubation at 37 °C for 45 min, and finally cooling to room temperature (22 °C). Alkylation was done by adding 15 μL of 0.5 M IAM into each solution followed by whirl mixing for 30 sec before incubation at 22 °C in the dark for 20 min. Protein precipitation was performed by adding ice-cold 80% ACN in LC-MS grade water (v/v), and whirl mixing for 30 sec before centrifugation for 15 min at 14,000 relative centrifugal force (rcf) in an Eppendorf 5415 R-model centrifuge (20 °C) (Hamburg, Germany). The supernatant was pipetted into a new tube and evaporated to dryness in a Speed Vac® SC110-model from Savant, Thermo Fisher Scientific (Waltham, MA, USA), followed by reconstitution in 100 μL 0.1% FA in LC-MS grade water (v/v). Aliquots of 10 μL of this solution were analyzed by the nanoLC-MS/MS platform.

For investigating protein binding, OT was spiked into human plasma and 500 μL was applied to 10 K Amicon® ultra centrifugal filters from Merck Millipore (Billerica, MA, USA). An aliquot of 20 μL of the filtrate was analyzed by the Bruker Easy nLC system (without AFFL) connected to a TSQ QuantivaTM triple quadrupole mass spectrometer from Thermo Scientific.

### Nasal spray experiment

Three healthy volunteers, one female and two males (age range 25–35) were asked to apply two puffs of OT nasal spray (8 IU OT, Syntocinon® from Sigma-Tau Pharmaceuticals, inc., Gaithersburg, MD, USA) in each nostril. Two post-application blood samples were drawn from each participant; one 5 min and another 20 min after the puffs of OT nasal spray were applied. Samples were immediately prepared and analyzed (4 aliquots of each sample was prepared and analyzed).

### Automatic filtration and filter back-flush (AFFL) solid phase extraction nanoLC tandem MS peptidomics platform

An EASY-nLC liquid chromatograph with an integrated 6 × 4 autosampler from Bruker (Billerica, MA, USA) was used as pump. Mobile phase A was 0.1% FA in LC-MS grade water (v/v), while Mobile phase B was 0.1% FA in LC-MS grade acetonitrile (ACN). The loading mobile phase composition was 0.1% FA in LC-MS grade water. The external 10-port valve from VICI (Schenkon, Switzerland) controlled by the MS-software was used in the AFFL system. See Fig. 1 for plumbing of the AFFL system. A Hitachi L-7100 HPLC pump (Chiyoda, Tokyo, Japan) in isocratic mode was used to back-flush the filter in the AFFL system with type 1 water. In position 1 (Fig. 1A), the sample passed through a stainless steel filter (1 μm porosity, 1/16″-screen, VICI) onto a 100 μm ID x 50 mm silica monolithic C18 SPE manufactured as described in^30^ (similar to Chromolith CapRod C18 capillary columns from Merck Millipore). In position 2 (Fig. 1B), two processes happened simultaneously; the filter is back-flushed, while oxytocin is back-flushed from the SPE column onto a 100 μm ID x 150 mm silica monolithic C18 separation column manufactured as described in^30^ (similar to Chromolith CapRod C18 capillary columns from Merck Millipore). A steel emitter, 30 μm ID x 40 mm, from Thermo Scientific, was connected to the end of the separation column by a 1/16″-standard steel internal union from VICI. A nanospray Flex^TM^ ion source (nanoESI) coupled to a Quantiva^TM^ triple quadrupole mass spectrometer from Thermo Scientific was used for detection of oxytocin in full MS- and tandem MS-mode (MS/MS).

### Liquid chromatography and mass spectrometry parameters

The 20 min gradient program was composed as follows: 20% B isocratic elution for 14 min, followed by an increase from 20 to 90% B in 2 min before isocratic elution at 90% B for 4 min. The flow rate during the chromatographic separation was 800 nL/min. The injection volume was 10 μL. The SPE was equilibrated with 4 μL 0.1% FA in LC-MS grade water at a constant flow rate of 3 μL/min, while the separation column was equilibrated with 5 μL 0.1% FA in LC-MS grade water at a flow rate of 3 μL/min before each injection. When running dozens of plasma samples consecutively, the 90% B washing step may be prolonged, to e.g. avoid lipid build-up in the system (which can lead to distorted peak shapes). The MS was operated in positive MS-mode and selected reaction monitoring (SRM) mode was used. The spray voltage was set to 1.6 kV. The precursor ions for native oxytocin and IS were *m/z* 1007.475 and *m/z* 1012.475, respectively. For oxytocin the product ions were *m/z* 285.125 with 38 V collision energy (CE), and *m/z* 723.225 with 30 V CE. For IS the product ions were *m/z* 290.125 with 38 V CE, and *m/z* 723.225 with 30 V CE. The precursor ions for reduced and alkylated (R/A) oxytocin and IS were *m/z* 1123.547 and *m/z* 1128.547, respectively. For R/A oxytocin the product ions were *m/z* 285.125 with 38 V CE, and *m/z* 839.302 with 30 V CE. For R/A IS the product ions were *m/z* 290.125 with 38 V CE and *m/z* 839.302 with 30 V CE. The Q1 and Q3 resolutions were both set to 1.2 FWHM, and the RF lens had a voltage of 185. A cycle time of 1 sec was used with 3 mTorr collision-induced dissociation (CID) gas. Argon was used as collision gas. In addition, 25 V source fragmentation energy was used together with 3 secs chrom filter.

### ELISA

Oxytocin ELISA kits were purchased from Arbor Assays and Cayman Chemical. 200 μL aliquots of pooled dog plasma were prepared using the R/A and PPT protocol described above. Samples were reconstituted in assay buffer, mixed together, and the resulting pool was measured at six different dilutions ranging from 10–100% of the fully concentrated sample.

To investigate the effect of the R/A procedure on detectable OT, 10 plasma samples were measured with both ELISA kits following solid phase extraction (SPE) or the R/A PPT protocol. SPE was performed using Oasis PRiME HLB cartridges (Waters Corporation, Milford, MA, USA) using a protocol previously validated in our laboratory. Samples were mixed 1:1 with 0.1% trifluoroacetic acid (TFA) and centrifuged at 14,000 RCF for 10 minutes. Cartridges were conditioned with 1 mL acetonitrile (HPLC grade), followed by 1 mL of 0.1% TFA before passing samples through the columns (gravity fed). Columns were then washed with 6 mL 0.1% TFA followed by OT elution with 95% acetonitrile, 0.1% TFA (v/v). Eluents were evaporated to dryness at 37 °C and then frozen at −20 °C until assayed, at which point samples were reconstituted in 250 μL assay-specific buffer. All samples were measured in duplicate.

### Data analysis and interpretation

Data analysis and interpretation were done using Xcalibur software version 3.0 from Thermo Scientific.

## Additional Information

**How to cite this article**: Brandtzaeg, O. K. *et al*. Proteomics tools reveal startlingly high amounts of oxytocin in plasma and serum. *Sci. Rep.* **6**, 31693; doi: 10.1038/srep31693 (2016).

## Supplementary Material

Supplementary Information
