# Supplementary material for: Proteomics tools reveal startlingly high amounts of oxytocin in plasma and serum
Source: Sci Rep. 2016 Aug 16;6:31693. doi: 10.1038/srep31693 (PMC4985690; doi:10.1038/srep31693)
Supplement: Supplementary Information [file srep31693-s1.doc]

**Supplementary data for:**

**Proteomics tools reveal startlingly high amounts of oxytocin in plasma and serum**

Ole Kristian Brandtzaeg^a^, Elin Johnsen^a^, Hanne Roberg-Larsen^a^, Knut Fredrik Seip^b^, Evan L. MacLean^c,d^, Laurence R. Gesquiere^e^, Siri Leknes^,f, g^, Elsa Lundanes^a^ and Steven Ray Wilson^a^*

^a^Department of Chemistry, University of Oslo, Post Box 1033, Blindern, NO-0315 Oslo, Norway

^b^School of Pharmacy, University of Oslo, PO Box 1068, Blindern, NO-0316, Oslo, Norway

^c^Department of Evolutionary Anthropology, Duke University, Durham, NC, 27708, USA

^d^School of Anthropology, University of Arizona, Tucson, AZ, 85721, USA

^e^Department of Biology, Duke University, Durham, NC, 27708, USA

^f^Department of Psychology, University of Oslo, PO Box 1094, Blindern, NO-0317, Oslo Norway

^g^The Intervention Centre, Oslo University Hospital, PO Box 4950, Nydalen, NO-0424 Oslo, Norway

*Corresponding author. Tel.: +47 970 10 953.

E-mail address: **stevenw@kjemi.uio.no** (S.R. Wilson).


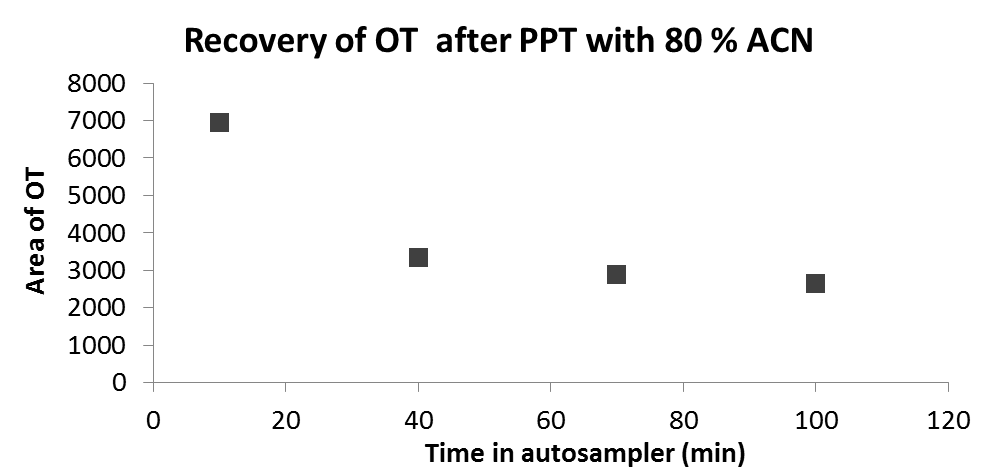


Figure SM 1 Recovery of OT after PPT with 80 % ACN. Plasma was spiked with 500 pg/mL OT and analyzed with the nano-AFFL-SPE-LC-MS/MS system after 10, 40, 70 and 100 minutes.


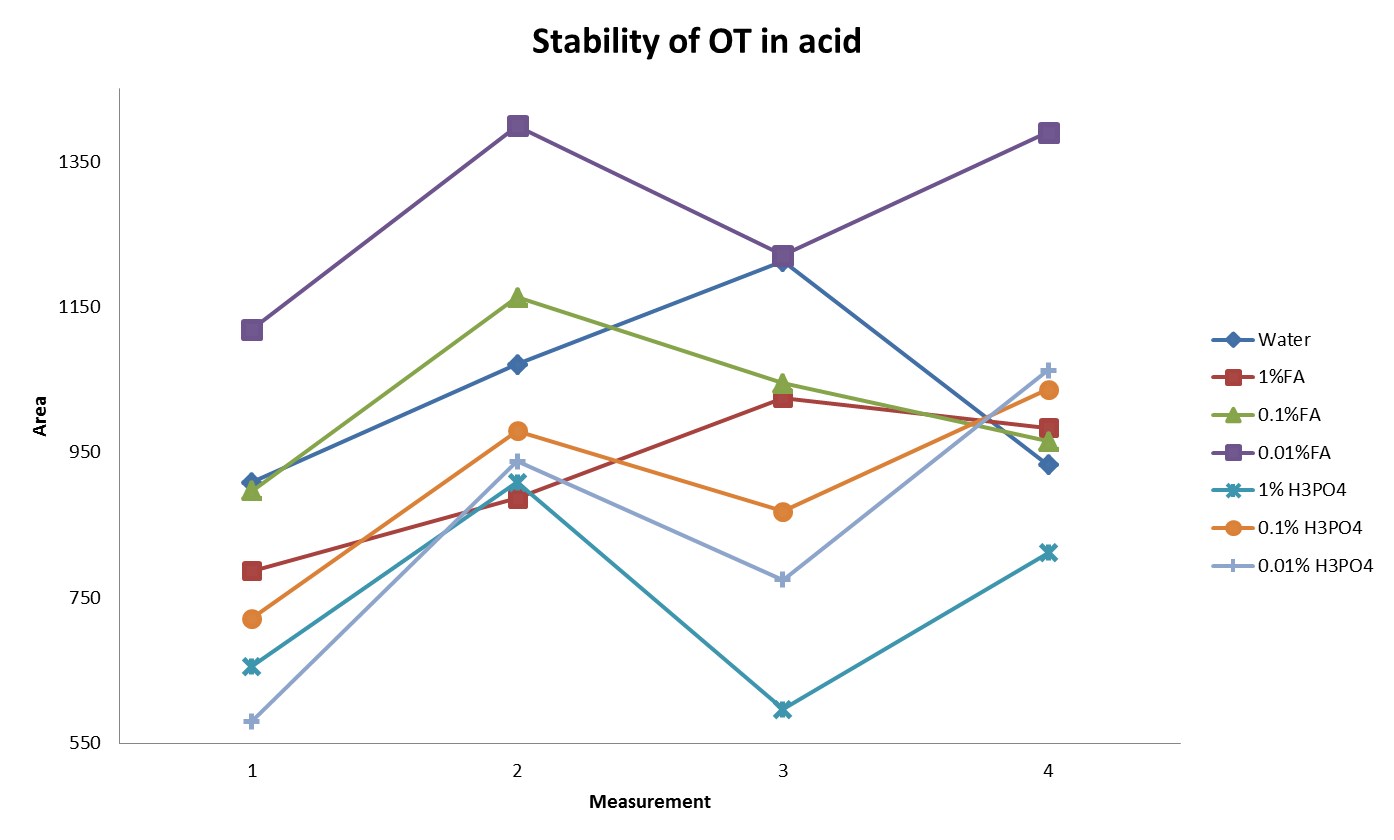


Figure SM 2: Stability of OT (100 ng/mL) spiked in water, formic acid (0.01-1 %) and H_3_PO_4_ (0.01-1 %). Measurement 1 was conducted 10 min after addition of acid, measurement 2 after 2 hours, measurement 3 after 3 hours and measurement 4 after 5 hours.


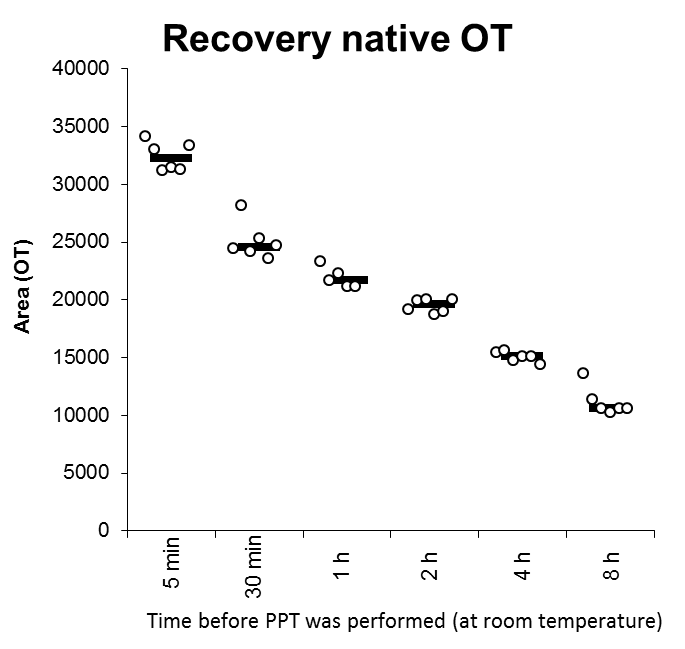


Figure SM 3 Recovery of native OT spiked into plasma (500 pg/mL) and incubated for 5 min – 6 hours in room temperature before PPT.


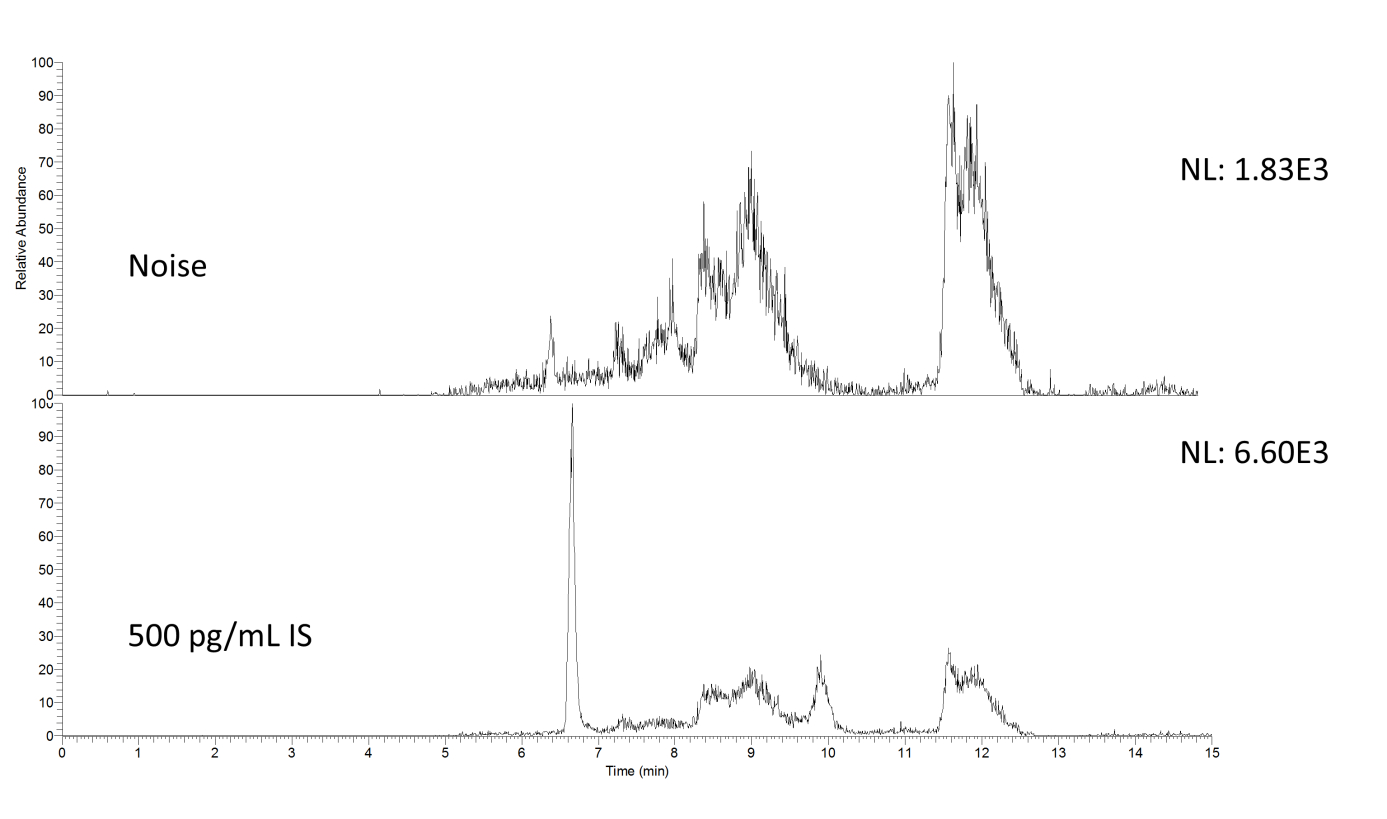


Figure SM 4 EIC (top, OT, m/z 1007→ 723, bottom, IS, m/z 1012 →723) analyzed on a Bruker easy nLC system without AFFL system (preliminary experiments). Mobile phase A was 0.1 % FA in H2O, while mobile phase 2 was 0.1 % FA in ACN. For elution a step gradient was used (0-10 min 0 -50 % B, 10-13 min 50-90 %B and 13-15 min 90 % B). Injection volume was 20 µL and flow rate was 800 nL/min


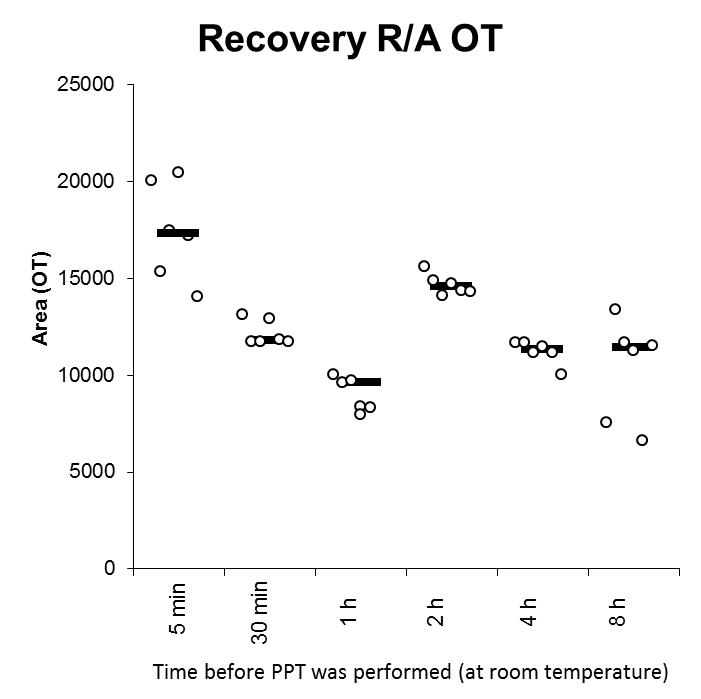


Figure SM 5 Recovery of R/A OT spiked into plasma (500 pg/mL) and incubated for 5 min – 6 hours in room temperature before PPT.


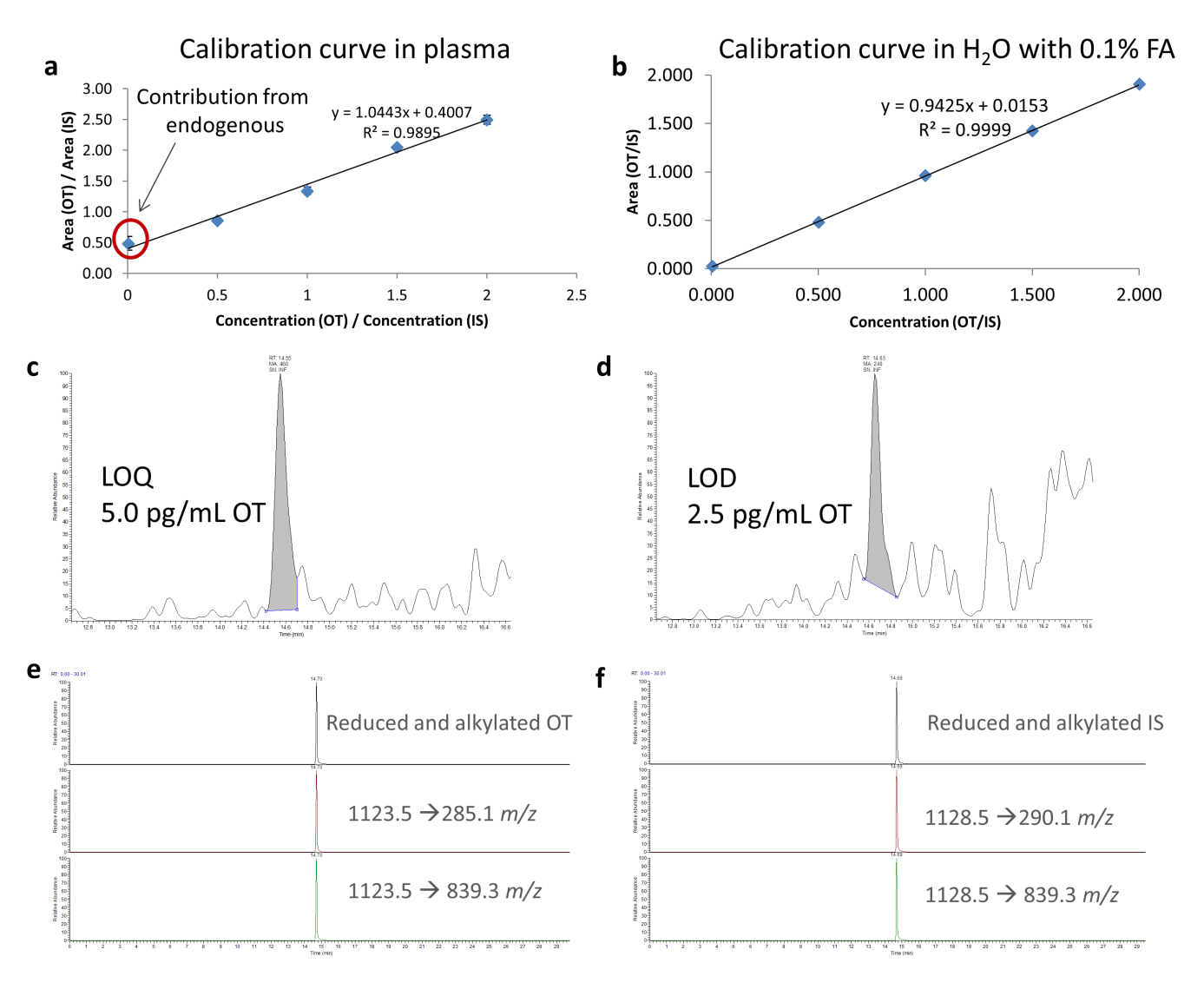


Figure SM 6 a: Calibration curve for reduced and alkylated OT in plasma. Pooled human plasma spiked with 5, 500, 1000, 1500 and 2000 pg/mL OT and 1000 pg/mL IS. b: Calibration curve for reduced and alkylated OT in water with 0.1% FA. Calibration standards were spiked with 5, 500, 1000, 1500 and 2000 pg/mL OT and 1000 pg/mL IS. c: Limit of quantification of 5 pg/mL reduced and alkylated oxytocin in water with 0.1% FA. d: Limit of detection was 2.5 pg/mL reduced and alkylated OT in water with 0.1% FA. e: Fragment transitions of reduced and alkylated oxytocin. f: Fragment transitions of reduced and alkylated IS.
